# Incidence of Multisystem Inflammatory Syndrome in Children Among US Persons Infected With SARS-CoV-2

**DOI:** 10.1001/jamanetworkopen.2021.16420

**Published:** 2021-06-10

**Authors:** Amanda B. Payne, Zunera Gilani, Shana Godfred-Cato, Ermias D. Belay, Leora R. Feldstein, Manish M. Patel, Adrienne G. Randolph, Margaret Newhams, Deepam Thomas, Reed Magleby, Katherine Hsu, Meagan Burns, Elizabeth Dufort, Angie Maxted, Michael Pietrowski, Allison Longenberger, Sally Bidol, Justin Henderson, Lynn Sosa, Alexandra Edmundson, Melissa Tobin-D’Angelo, Laura Edison, Sabrina Heidemann, Aalok R. Singh, John S. Giuliano, Lawrence C. Kleinman, Keiko M. Tarquinio, Rowan F. Walsh, Julie C. Fitzgerald, Katharine N. Clouser, Shira J. Gertz, Ryan W. Carroll, Christopher L. Carroll, Brooke E. Hoots, Carrie Reed, F. Scott Dahlgren, Matthew E. Oster, Timmy J. Pierce, Aaron T. Curns, Gayle E. Langley, Angela P. Campbell, and the MIS-C Incidence Authorship Group, Neha Balachandran, Thomas S. Murray, Cole Burkholder, Troy Brancard, Jenna Lifshitz, Dylan Leach, Ian Charpie, Cory Tice, Susan E. Coffin, Dana Perella, Kaitlin Jones, Kimberly L. Marohn, Phoebe H. Yager, Neil D. Fernandes, Heidi R. Flori, Monica L. Koncicki, Karen S. Walker, Maria Cecilia Di Pentima, Simon Li, Steven M. Horwitz, Sunanda Gaur, Dennis C. Coffey, Ilana Harwayne-Gidansky, Saul R. Hymes, Neal J. Thomas, Kate G. Ackerman, Jill M. Cholette

**Affiliations:** 1 COVID-19 Response Team, Centers for Disease Control and Prevention, Atlanta, Georgia; 2 Department of Anesthesiology, Critical Care, and Pain Medicine, Boston Children’s Hospital, Boston, Massachusetts; 3 Department of Anesthesia and Pediatrics, Harvard Medical School, Boston, Massachusetts; 4 New Jersey Department of Health, Trenton; 5 Epidemic Intelligence Service, Centers for Disease Control and Prevention, Atlanta, Georgia; 6 Massachusetts Department of Health, Boston; 7 New York State Department of Health, Albany; 8 Philadelphia Department of Public Health, Philadelphia, Pennsylvania; 9 Pennsylvania Department of Health, Harrisburg; 10 Michigan Department of Health and Human Services, Lansing; 11 Enteric and Respiratory Illnesses Epidemiology Unit, Surveillance and Infectious Disease Epidemiology Section, Communicable Disease Division, Michigan Department of Health and Human Services, Lansing; 12 Connecticut Department of Public Health, Hartford; 13 Council of State and Territorial Epidemiologists, Atlanta, Georgia; 14 Acute Disease Epidemiology Section, Georgia Department of Public Health, Atlanta; 15 Department of Pediatrics, Division of Pediatric Critical Care Medicine, Central Michigan University, Detroit; 16 Pediatric Critical Care Division, Maria Fareri Children’s Hospital at Westchester Medical Center, Westchester, New York; 17 New York Medical College, Valhalla; 18 Department of Pediatrics, Division of Critical Care, Yale University School of Medicine, New Haven, Connecticut; 19 Department of Pediatrics, Division of Population Health, Quality, and Implementation Sciences, Rutgers Robert Wood Johnson Medical School, New Brunswick, New Jersey; 20 Division of Critical Care Medicine, Department of Pediatrics, Emory University School of Medicine, Children’s Healthcare of Atlanta, Atlanta, Georgia; 21 Department of Pediatrics, Division of Pediatric Cardiology, Children’s Hospital of New Jersey, Newark Beth Israel, Newark; 22 Division of Critical Care, Department of Anesthesiology and Critical Care, University of Pennsylvania Perelman School of Medicine, Philadelphia; 23 Division of Hospital Medicine, Department of Pediatrics, Hackensack University Medical Center, Hackensack, New Jersey; 24 Division of Pediatric Critical Care, Department of Pediatrics, St Barnabas Medical Center, Livingston, New Jersey; 25 Division of Pediatric Critical Care Medicine, MassGeneral Hospital for Children, Harvard Medical School, Boston, Massachusetts; 26 Division of Critical Care, Connecticut Children’s, Hartford; 27 Department of Pediatrics, Infectious Disease and Global Health, Yale School of Medicine, New Haven, Connecticut; 28 Emory University School of Medicine, Children’s Healthcare of Atlanta, Atlanta, Georgia; 29 Baystate Children’s Hospital, Springfield, Massachusetts; 30 MassGeneral Hospital for Children, Harvard Medical School, Boston, Massachusetts; 31 University of Michigan CS Mott Children’s Hospital, Ann Arbor; 32 St Christopher’s Hospital for Children, Philadelphia, Pennsylvania; 33 Cooper University Hospital, Camden, New Jersey; 34 Goryeb Children’s Hospital, Morristown, New Jersey; 35 Bristol-Myers Squibb Children’s Hospital, New Brunswick, New Jersey; 36 The Valley Hospital, Ridgewood, New Jersey; 37 Stony Brook University Hospital, Stony Brook, New York; 38 Penn State Children’s Hospital, Hershey, Pennsylvania; 39 Golisano Children’s Hospital, Rochester, New York

## Abstract

**Importance:**

Multisystem inflammatory syndrome in children (MIS-C) is associated with recent or current SARS-CoV-2 infection. Information on MIS-C incidence is limited.

**Objective:**

To estimate population-based MIS-C incidence per 1 000 000 person-months and to estimate MIS-C incidence per 1 000 000 SARS-CoV-2 infections in persons younger than 21 years.

**Design, Setting, and Participants:**

This cohort study used enhanced surveillance data to identify persons with MIS-C during April to June 2020, in 7 jurisdictions reporting to both the Centers for Disease Control and Prevention national surveillance and to Overcoming COVID-19, a multicenter MIS-C study. Denominators for population-based estimates were derived from census estimates; denominators for incidence per 1 000 000 SARS-CoV-2 infections were estimated by applying published age- and month-specific multipliers accounting for underdetection of reported COVID-19 case counts. Jurisdictions included Connecticut, Georgia, Massachusetts, Michigan, New Jersey, New York (excluding New York City), and Pennsylvania. Data analyses were conducted from August to December 2020.

**Exposures:**

Race/ethnicity, sex, and age group (ie, ≤5, 6-10, 11-15, and 16-20 years).

**Main Outcomes and Measures:**

Overall and stratum-specific adjusted estimated MIS-C incidence per 1 000 000 person-months and per 1 000 000 SARS-CoV-2 infections.

**Results:**

In the 7 jurisdictions examined, 248 persons with MIS-C were reported (median [interquartile range] age, 8 [4-13] years; 133 [53.6%] male; 96 persons [38.7%] were Hispanic or Latino; 75 persons [30.2%] were Black). The incidence of MIS-C per 1 000 000 person-months was 5.1 (95% CI, 4.5-5.8) persons. Compared with White persons, incidence per 1 000 000 person-months was higher among Black persons (adjusted incidence rate ratio [aIRR], 9.26 [95% CI, 6.15-13.93]), Hispanic or Latino persons (aIRR, 8.92 [95% CI, 6.00-13.26]), and Asian or Pacific Islander (aIRR, 2.94 [95% CI, 1.49-5.82]) persons. MIS-C incidence per 1 000 000 SARS-CoV-2 infections was 316 (95% CI, 278-357) persons and was higher among Black (aIRR, 5.62 [95% CI, 3.68-8.60]), Hispanic or Latino (aIRR, 4.26 [95% CI, 2.85-6.38]), and Asian or Pacific Islander persons (aIRR, 2.88 [95% CI, 1.42-5.83]) compared with White persons. For both analyses, incidence was highest among children aged 5 years or younger (4.9 [95% CI, 3.7-6.6] children per 1 000 000 person-months) and children aged 6 to 10 years (6.3 [95% CI, 4.8-8.3] children per 1 000 000 person-months).

**Conclusions and Relevance:**

In this cohort study, MIS-C was a rare complication associated with SARS-CoV-2 infection. Estimates for population-based incidence and incidence among persons with infection were higher among Black, Hispanic or Latino, and Asian or Pacific Islander persons. Further study is needed to understand variability by race/ethnicity and age group.

## Key Points

**Question:**

What was the incidence of multisystem inflammatory syndrome in children (MIS-C) among persons with SARS-CoV-2 infection in the US during April to June 2020?

**Findings:**

In this cohort study of 248 persons with MIS-C, MIS-C incidence was 5.1 persons per 1 000 000 person-months and 316 persons per 1 000 000 SARS-CoV-2 infections in persons younger than 21 years. Incidence was higher among Black, Hispanic or Latino, and Asian or Pacific Islander persons compared with White persons and in younger persons compared with older persons.

**Meaning:**

These findings suggest that MIS-C was a rare complication of SARS-CoV-2 infection; further study is needed to understand why MIS-C incidence varied by race/ethnicity and age group.

## Teaser

This cohort study examines the incidence of multisystem inflammatory syndrome in children among persons in the US with SARS-CoV-2 infection.

## Introduction

SARS-CoV-2 infections in most children result in less severe COVID-19 than infections in adults.^1,2,3,4^ However, a subset of children present with severe multisystem inflammation associated with current or recent SARS-CoV-2 infection or COVID-19 exposure in the weeks before.^5,6,7,8,9,10^ In the United States, this condition has been termed *multisystem inflammatory syndrome in children* (MIS-C).^11^ While data have been published on the epidemiological characteristics and clinical spectrum of MIS-C,^5,12,13,14,15,16,17^ information on incidence of MIS-C is limited. One report by Dufort et al^5^ describing MIS-C in New York state estimated the incidence to be 2 cases per 100 000 persons younger than 21 years during March 1 to May 10, 2020. A study from New York City by Lee et al^18^ estimated the incidence of MIS-C to be 11.4 per 100 000 persons younger than 20 years during March 1 to June 30, 2020. Available data suggest that MIS-C incidence may vary by certain characteristics, with MIS-C occurring disproportionately among Black and Hispanic or Latino persons and occurrence varying by age, sex, and geographic location.^19^

MIS-C has a strong epidemiological and laboratory association with SARS-CoV-2 infection,^19^ and it could be expected that higher MIS-C incidence among certain demographic groups might reflect rates of COVID-19 cases in the total population. However, an important question is whether certain racial and ethnic groups might be disproportionately represented among persons with MIS-C relative to overall SARS-CoV-2 infections. In other words, what is the incidence of MIS-C across groups using a denominator representing total SARS-CoV-2 infections?

The ability to estimate MIS-C incidence relative to overall SARS-CoV-2 infections has been restricted by the limited ability to estimate the number of SARS-CoV-2 infections based on reported COVID-19 case counts. Multiple factors contributed to underdetection of overall SARS-CoV-2 infections in the US, including limitations in testing availability and reporting practices, assay sensitivity, care-seeking behaviors, and underrecognition of mild or asymptomatic infection.^8^ However, as more information became available, multipliers accounting for underascertainment were published to adjust nationally reported COVID-19 case counts to better reflect the true number of SARS-CoV-2 infections.^8^

Our objectives were to estimate the population-based incidence of MIS-C per 1 000 000 person-months and to estimate incidence of MIS-C per 1 000 000 SARS-CoV-2 infections in persons younger than 21 years, estimating overall and by jurisdiction, race/ethnicity, sex, and age group.

## Methods

The study was approved by the central institutional review board at Boston Children’s Hospital and determined to meet the requirement of public health surveillance as defined in 45 CFR 46.102(I)(2) at Boston Children’s Hospital and the US Centers for Disease Control and Prevention (CDC) under a waiver of consent. This study follows the Strengthening the Reporting of Observational Studies in Epidemiology (STROBE) reporting guideline.

### Identifying Persons With MIS-C

On May 14, 2020, the CDC initiated national surveillance for MIS-C with publication of an official health advisory, requesting health care practitioners report suspected MIS-C cases to local, state, and territorial public health authorities.^11^ A second MIS-C surveillance system, the Overcoming COVID-19 study, included 60 participating hospitals during the study period (the first hospitalization date included was March 16, 2020) in a network previously established by the CDC for influenza surveillance.^15^

We enhanced surveillance by matching and deduplicating persons reported to the national surveillance system (a passive surveillance system) and the Overcoming COVID-19 study (an active surveillance system), to attempt to identify all reported MIS-C cases during April 1 to June 30, 2020, from jurisdictions reporting persons to the CDC’s national surveillance and from hospitals reporting to Overcoming COVID-19. Of 27 jurisdictions reporting, the 8 that reported at least 10 persons with MIS-C to Overcoming COVID-19 were approached for participation and 7 jurisdictions agreed: Connecticut, Georgia, Massachusetts, Michigan, New Jersey, New York (excluding New York City), and Pennsylvania. Catchment areas were defined by entire states (except for New York) and persons reported by either or both systems were included.

Health departments reported persons younger than 21 years to the CDC’s national surveillance using the CDC MIS-C Case Report Form (CRF)^20^ with the case definition of clinically severe illness necessitating hospitalization with fever, elevation of inflammatory markers, involvement of 2 or more organ systems, no alternative plausible diagnosis, and current or recent SARS-CoV-2 infection, as identified by a positive reverse transcription–polymerase chain reaction (RT-PCR) test, serological test, or antigen test, or exposure to a suspected or confirmed COVID-19 case within 4 weeks prior to symptom onset (eAppendix 1 in the Supplement).^11^ Persons with MIS-C reported to Overcoming COVID-19 were identified through prospective and retrospective surveillance at participating hospitals.^15^ Children with MIS-C reported in Overcoming COVID-19 met the CDC case definition during April 1 to May 31, 2020; however, after June 1, 2020, persons were excluded if test results were negative or unknown by RT-PCR and antibody testing or if they did not have SARS-CoV-2 laboratory testing (ie, epidemiologic link with prior exposure to a COVID-19 case was insufficient to meet MIS-C criteria) (eAppendix 2 in the Supplement).

We matched and deduplicated case reports between the CDC’s national surveillance database and the Overcoming COVID-19 database to capture unique persons for each jurisdiction. Matching was performed using admitting hospital, date of birth, admission date, and 4-digit zip code of residence (Overcoming COVID-19 limited collection to the first 4 digits). We adjudicated discrepancies with submitting jurisdictions. Age group (ie, ≤5, 6-10, 11-15, and 16-20 years) was defined by age at admission (or, if not available, age at symptom onset). We assigned race/ethnicity group based on race and ethnicity reported on the CRF, which was obtained from hospital medical records as documented at the time of hospitalization.

### Denominator for Incidence per Person-Month

We estimated denominators for the calculation of MIS-C incidence per 1 000 000 person-months based on US Census bridged-race population 2019 estimates.^21^ Estimates by jurisdiction, age group, sex, and race/ethnicity were used. We multiplied population estimates by 3 months to estimate the person-months at risk during the study period.

### Denominator for Incidence per SARS-CoV-2 Infection

We estimated denominators for MIS-C incidence per SARS-CoV-2 infection among persons younger than 21 years based on COVID-19 cases reported during March 1 to May 31, 2020, by each jurisdiction. This period was selected to account for the approximate 4-week temporal lag between SARS-CoV-2 infection and MIS-C development.^5,15,19^ To account for factors related to underascertainment of SARS-CoV-2 infections, we adapted and applied recently published age- and month-specific multipliers^8^ to reported COVID-19 case counts to estimate total number of SARS-CoV-2 infections (eTable 1 in the Supplement). The denominators of SARS-CoV-2 infections among persons younger than 21 years were calculated from reported COVID-19 cases, which varied by jurisdiction by month (eFigure 1 in the Supplement). The multipliers used to estimate the number of underlying SARS-CoV-2 infections decreased during March through May (eTable 1 in the Supplement).

### Statistical Analysis

We estimated overall MIS-C incidence per 1 000 000 person-months by dividing the number of reported MIS-C cases by the estimated per-month population. Similarly, we estimated overall MIS-C incidence per SARS-CoV-2 infection by dividing the number of reported MIS-C cases by the estimated number of SARS-CoV-2 infections among persons younger than 21 years. We obtained 95% CIs using univariate Poisson regression^22^ with offsets from population estimates or number of SARS-CoV-2 infections, as appropriate. We estimated stratum-specific adjusted MIS-C incidence using multivariable Poisson regression^22^ with jurisdiction, age group, sex, and race/ethnicity included in the model. Adjusted incidence rate ratios (aIRRs) were used to compare differences between groups, with 95% CIs not overlapping 1.0 being considered statistically significant. Significance tests were 2-sided. A range of incidence per 1 000 000 SARS-CoV-2 infection estimates were produced based on reported COVID-19 cases, and the point estimate and 95% CIs around the age- and month-specific multipliers were used to account for underdetection of SARS-CoV-2.

We categorized race/ethnicity based on categories used in the US Census bridged-race population estimates^21^: Black, White, Asian or Pacific Islander, or American Indian or Alaska Native race and Hispanic or Latino ethnicity. For this analysis, we used the following nonoverlapping categories: Hispanic or Latino (regardless of race), or Black, White, Asian or Pacific Islander, and American Indian or Alaska Native (each of non-Hispanic or unknown ethnicity). We used multiple imputation to impute race/ethnicity of persons with COVID-19 with unknown race or race originally reported as other or multiple (16 129 persons [48.6%]) based on the distribution of race/ethnicity according to the bridged-race jurisdiction population estimates.^21^ Iterations of imputation were combined to capture both the within- and between-iteration variance.^23^ While some iterations of imputation assigned race/ethnicity as American Indian or Alaska Native and there were 25 American Indian or Alaska Native persons with COVID-19 reported by jurisdictions, no American Indian or Alaska Native persons were reported with MIS-C, thus incidence estimates for American Indian or Alaska Native persons were not reported. Persons with MIS-C with unknown race or race originally reported as other or multiple (32 persons [12.9%]) were excluded from analyses involving race/ethnicity, including adjusted analyses.

We conducted all analyses using SAS version 9.4. Data analyses were conducted from August to December 2020.

## Results

### MIS-C Identified by Enhanced Surveillance

During April to June 2020, 248 persons with MIS-C were reported in participating jurisdictions (Table 1). New Jersey reported the largest number, followed by Massachusetts, New York, Michigan, Pennsylvania, Connecticut, and Georgia. More than half of persons with MIS-C were male (133 persons [53.6%]); 96 persons (38.7%) were Hispanic or Latino, and 75 persons (30.2%) were Black. Median (interquartile range) age at MIS-C onset was 8 (4-13) years. Distribution of demographic characteristics of persons with MIS-C varied by jurisdiction (eFigure 2 in the Supplement).

**Table 1.** Distribution of Persons With Multisystem Inflammatory Syndrome in Children Reported to the Centers for Disease Control and Prevention’s National Surveillance or the Overcoming COVID-19 Study From Select Jurisdictions During April to June 2020, by Jurisdiction, Race/Ethnicity, Sex, and Age Group

Characteristic	No.			Total deduplicated persons, No. (%)
	National surveillance	Overcoming COVID-19	Both systems	
Total	98	56	94	248
Jurisdiction				
Connecticut	2	5	12	19 (7.7)
Georgia	3	2	9	14 (5.6)
Massachusetts	15	7	26	48 (19.4)
Michigan	6	9	13	28 (11.3)
New Jersey	30	23	18	71 (28.6)
New York^a^	20	10	11	41 (16.5)
Pennsylvania	22	0	5	27 (10.9)
Race/ethnicity				
Hispanic or Latino	43	20	33	96 (38.7)
Black	28	10	37	75 (30.2)
White	15	11	8	34 (13.7)
Asian or Pacific Islander	3	5	3	11 (4.4)
American Indian or Alaska Native	0	0	0	0
Unknown, other, or multiple	9	10	13	32 (12.9)
Sex				
Female	47	28	40	115 (46.4)
Male	51	28	54	133 (53.6)
Age group, y				
≤5	30	26	27	83 (33.5)
6-10	33	11	35	79 (31.9)
11-15	21	8	22	51 (20.6)
16-20	14	11	10	35 (14.1)

^a^ Excludes New York City.

### Incidence of MIS-C per 1 000 000 Person-Months

Overall incidence of MIS-C during the study period was 5.1 (95% CI, 4.5-5.8) persons per 1 000 000 person-months. Stratum-specific adjusted incidence of MIS-C per 1 000 000 person-months varied by jurisdiction, race/ethnicity, and age group (Table 2). The adjusted incidence ranged from 1.0 (95% CI, 0.5-1.8) persons per 1 000 000 person-months in Georgia to 8.5 (95% CI, 6.1-12.0) persons per 1 000 000 person-months in Massachusetts. The highest incidence was among Black persons (9.6 [95% CI, 7.6-12.2] persons per 1 000 000 person-months) and persons of Hispanic or Latino ethnicity (9.3 [95% CI, 7.4-11.7] persons per 1 000 000 person-months). Compared with White persons, incidence was approximately 9-fold higher among Black persons (aIRR, 9.26 [95% CI, 6.15-13.93]) and Hispanic or Latino persons (aIRR, 8.92 [95% CI, 6.00-13.26]) and approximately 3-fold higher among Asian or Pacific Islander persons (aIRR, 2.94 [95% CI, 1.49-5.82]). Incidence did not differ significantly by sex (aIRR, 1.10 [95% CI, 0.84-1.43]). Incidence was highest among children aged 5 years or younger (4.9 [95% CI, 3.7-6.6] children per 1 000 000 person-months) and children aged 6 to 10 years (6.3 [95% CI, 4.8-8.3] children per 1 000 000 person-months) (Table 2). Compared with children aged 5 years or younger, persons aged 16 to 20 years had significantly lower incidence (aIRR, 0.48 [95% CI, 0.31-0.74]).

**Table 2.** Stratum-Specific Incidence of MIS-C per 1 000 000 Person-Months During April to June 2020 in Select Jurisdictions by Jurisdiction, Race/Ethnicity, Sex, and Age Group

Characteristic	No.		Incidence per 1 000 000 person-months (95% CI)		Adjusted incidence rate ratio (95% CI)^a^^,^^b^
	Persons with MIS-C	Population aged <21 y	Unadjusted	Adjusted^a^^,^^b^	
Jurisdiction					
Connecticut	19	881 871	7.2 (4.6-11.3)	6.2 (3.8-10.1)	1 [Reference]
Georgia	14	2 945 570	1.6 (0.9-2.7)	1.0 (0.5-1.8)	0.16 (0.07-0.33)
Massachusetts	48	1 660 823	9.6 (7.3-12.8)	8.5 (6.1-12)	1.37 (0.79-2.40)
Michigan	28	2 541 087	3.7 (2.5-5.3)	3.6 (2.4-5.6)	0.59 (0.32-1.09)
New Jersey	71	2 255 747	10.5 (8.3-13.2)	7.5 (5.6-10)	1.20 (0.71-2.03)
New York^c^	41	2 789 675	4.9 (3.6-6.7)	4.5 (3.1-6.5)	0.72 (0.41-1.28)
Pennsylvania	27	3 143 391	2.9 (2.0-4.2)	3.1 (2.1-4.7)	0.50 (0.27-0.92)
Race/ethnicity^b^					
White	34	9 730 939	1.2 (0.8-1.6)	1.0 (0.7-1.5)	1 [Reference]
Black	75	2 868 167	8.7 (7-10.9)	9.6 (7.6-12.2)	9.26 (6.15-13.93)
Hispanic or Latino	96	2 646 523	12.1 (9.9-14.8)	9.3 (7.4-11.7)	8.92 (6.00-13.26)
Asian or Pacific Islander	11	914 796	4.0 (2.2-7.2)	3.1 (1.7-5.6)	2.94 (1.49-5.82)
Sex					
Female	115	7 942 364	4.8 (4.0-5.8)	3.9 (3.1-5.0)	1 [Reference]
Male	133	8 275 800	5.4 (4.5-6.3)	4.3 (3.4-5.5)	1.10 (0.84-1.43)
Age group, y					
≤5	83	4 315 512	6.4 (5.2-8.0)	4.9 (3.7-6.6)	1 [Reference]
6-10	79	3 741 919	7.0 (5.6-8.8)	6.3 (4.8-8.3)	1.28 (0.92-1.78)
11-15	51	3 943 666	4.3 (3.3-5.7)	3.8 (2.8-5.3)	0.77 (0.53-1.13)
16-20	35	4 217 067	2.8 (2.0-3.9)	2.4 (1.6-3.5)	0.48 (0.31-0.74)

Abbreviation: MIS-C, multisystem inflammatory syndrome in children.

^a^ Adjusted using Poisson regression, with jurisdiction, race/ethnicity, sex, and age group in the model.

^b^ A total of 32 persons with MIS-C with unknown or other race/ethnicity were excluded from analyses involving race/ethnicity, including adjusted estimates.

^c^ Excludes New York City.

### Incidence of MIS-C per 1 000 000 SARS-CoV-2 Infections

Overall incidence of MIS-C was 316 (95% CI, 278-357) persons per 1 000 000 SARS-CoV-2 infections. Adjusted estimates ranged from 627 (95% CI, 405-969) persons per 1 000 000 SARS-CoV-2 infections in Michigan to 138 (95% CI, 96-198) persons per 1 000 000 SARS-CoV-2 infections in New York (Table 3). Compared with White persons, MIS-C incidence estimates per 1 000 000 SARS-CoV-2 infections were higher among Black persons (aIRR, 5.62 [95% CI, 3.68-8.60]), Hispanic or Latino persons (aIRR, 4.26 [95% CI, 2.85-6.38]), and Asian or Pacific Islander persons (aIRR, 2.88 [95% CI, 1.42-5.83]). There was no significant difference between males and females (aIRR, 1.04 [95% CI, 0.80-1.37]). Compared with children aged 5 years or younger, MIS-C incidence per 1 000 000 SARS-CoV-2 infections was higher among children aged 6 to 10 years (aIRR, 1.38 [95% CI, 0.99-1.93]) and lower among children aged 11 to 15 years (aIRR, 0.50 [95% CI, 0.34-0.74]) and persons aged 16 to 20 years (aIRR, 0.37 [95% CI, 0.24-0.57]). eTable 2 in the Supplement provides the adjusted MIS-C incidence per 1 000 000 persons with reported COVID-19 and outlines the range of estimates for MIS-C per 1 000 000 SARS-CoV-2 infections using the point estimate and 95% CIs around the month- and age-specific multipliers.^8^

**Table 3.** Stratum-Specific Incidence of MIS-C per 1 000 000 SARS-CoV-2 Infections During April to June 2020 in Select Jurisdictions by Jurisdiction, Race/Ethnicity, Sex, and Age Group

Characteristic	No.			MIS-C incidence per 1 000 000 SARS-CoV-2 infections (95% CI)^a^		Adjusted MIS-C incidence rate ratio (95% CI)^a^^,^^c^^,^^d^
	Reported persons with MIS-C	Reported persons with COVID-19	Estimated SARS-CoV-2 infections^b^	Unadjusted	Adjusted^c^^,^^d^	
Jurisdiction						
Connecticut	19	2103	48 270	394 (251-617)	358 (219-585)	1 [Reference]
Georgia	14	2840	38 284	366 (217-617)	246 (136-444)	0.69 (0.33-1.43)
Massachusetts	48	6006	102 729	467 (352-620)	352 (248-498)	0.98 (0.56-1.72)
Michigan	28	2239	35 961	779 (538-1128)	627 (405-969)	1.75 (0.94-3.26)
New Jersey^e^	71	6862	180 091	394 (312-497)	331 (248-443)	0.92 (0.54-1.57)
New York^f^	41	9524	313 012	131 (96-178)	138 (96-198)	0.38 (0.22-0.69)
Pennsylvania	27	3590	67 114	402 (276-587)	355 (234-540)	0.99 (0.54-1.82)
Race/Ethnicity^c^						
White	34	4667	106 053	87 (62-122)	110 (77-156)	1 [Reference]
Black	75	3624	67 154	612 (485-772)	616 (481-790)	5.62 (3.68-8.6)
Hispanic or Latino	96	7225	128 174	458 (375-561)	467 (371-588)	4.26 (2.85-6.38)
Asian or Pacific Islander	11	581	11 599	283 (155-518)	315 (169-589)	2.88 (1.42-5.83)
Sex^g^						
Female	115	16 487	368 368	312 (260-375)	309 (240-399)	1 [Reference]
Male	133	16 489	410 507	324 (273-384)	323 (252-413)	1.04 (0.80-1.37)
Age group, y						
≤5	83	4707	176 801	469 (379-582)	444 (333-591)	1 [Reference]
6-10	79	3805	149 212	529 (425-660)	613 (464-811)	1.38 (0.99-1.93)
11-15	51	6230	259 503	197 (149-259)	224 (160-312)	0.50 (0.34-0.74)
16-20	35	18 422	199 947	175 (126-244)	164 (110-243)	0.37 (0.24-0.57)

Abbreviation: MIS-C, multisystem inflammatory syndrome in children.

^a^ Reflects estimate after imputation of race/ethnicity for 16 129 persons with COVID-19 reported from jurisdictions.

^b^ SARS-CoV-2 infections estimated by applying age- and month-specific multipliers to reported COVID-19 case counts.

^c^ Adjusted using Poisson regression, with jurisdiction, race/ethnicity, sex, and age group in the model.

^d^ A total of 32 persons with MIS-C with other or unknown race/ethnicity were excluded from analyses involving race/ethnicity, including adjusted estimates.

^e^ New Jersey suppressed stratum-specific reported COVID-19 case counts of fewer than 5; these were assumed to be 1 for analysis.

^f^ Excludes New York City.

^g^ A total of 188 reported persons with COVID-19 with other or unknown sex were excluded from adjusted analyses.

## Discussion

The findings of this cohort study suggest that MIS-C is a rare complication of SARS-CoV-2 infection among persons younger than 21 years. Adjusted incidence estimates were approximately 1 to 10 cases per 1 000 000 person-months, with estimates varying by race/ethnicity, age group, and jurisdiction. To assess risk among persons infected with SARS-CoV-2, we estimated incidence of MIS-C per 1 000 000 SARS-CoV-2 infections. Incidence was consistently higher among Black persons, Hispanic or Latino persons, and Asian or Pacific Islander persons compared with White persons. MIS-C incidence per 1 000 000 person-months among Black persons and Hispanic or Latino persons was approximately 9-fold higher, and incidence among Asian or Pacific Islander persons was approximately 3-fold higher compared with White persons. Additionally, incidence of MIS-C per 1 000 000 SARS-CoV-2 infections among Black persons, Hispanic or Latino persons, and Asian or Pacific Islander persons was significantly higher than incidence among White persons. Similarly, MIS-C incidence was consistently significantly lower among persons aged 16 to 20 years compared with children aged 5 years or younger.

Appropriate incidence estimation involves careful estimation of the numerator (in this instance, persons with MIS-C) and denominator (jurisdiction population and number of SARS-CoV-2 infections). These analyses are strengthened by use of MIS-C reporting through enhanced surveillance. Active surveillance in the Overcoming COVID-19 study, although robust in case identification and data collection, is limited to the number of sites in which the surveillance is feasible, making broad catchment coverage less achievable. Passive surveillance, although allowing a broader catchment area, is limited because of reliance on timely reporting from jurisdictions. Enhanced surveillance leveraged active and passive surveillance systems to increase MIS-C case identification and allowed for estimation of incidence per population using readily obtainable jurisdiction-specific estimates. Furthermore, applying previously published multipliers^8^ to account for underdetection of SARS-CoV-2 infections to jurisdiction-reported stratum-specific COVID-19 case counts allowed for estimation of incidence per SARS-CoV-2 infection.

Persons with MIS-C in our study are similar in characteristics to persons previously reported. A systematic review of studies from the United States and Europe^19^ found 31% to 62% of reported persons with MIS-C were Black or Afro-Caribbean and 36% to 39% were Hispanic or Latino, 43% to 73% were male, and the median age at onset was 7.3 to 10 years across studies. Our MIS-C population-based incidence estimates of approximately 1 to 8.5 persons with MIS-C per 1 000 000 person-months across jurisdictions are similar to the estimate described for New York state over a 2.5-month time frame.^5^ The population-based incidence estimates per 1 000 000 person-months would roughly translate to 0.1 to 0.85 persons with MIS-C per 100 000 population per month, or 1.2 to 10.2 persons with MIS-C per 100 000 population per year, although we cannot assume these estimates were constant over a year. To put these findings into context, recent annual US Kawasaki disease–associated hospitalization rates for children younger than 5 years were approximately 18 to 20 persons per 100 000 population, depending on the data source.^24^ The estimated MIS-C incidence per 1 000 000 SARS-CoV-2 infections described in this report was lower than that reported in an international cohort that included incidence estimates reported per COVID-19 case identified through administrative records.^25^ Lower incidence described in our analysis may be explained, in part, by underdetection of SARS-CoV-2 infections, highlighting the importance of adjusting reported COVID-19 case counts for underascertainment.

One study in New York City reported a higher per-population burden of MIS-C among Black and Hispanic or Latino persons compared with White persons.^18^ However, it has remained unclear if the disproportionately higher percentages of Black and Hispanic or Latino persons with MIS-C compared with White persons was associated with the increased burden of COVID-19 among these populations.^24^ Our findings suggest that there was an increased incidence of MIS-C per SARS-CoV-2 infection among Black and Hispanic or Latino persons after accounting for increased burden of COVID-19. The cause of this increased incidence requires further investigation; however, the inequitable distribution of social determinants of health, such as social, economic, and environmental conditions, has been recognized as a contributor associated with persistent and pervasive health disparities.^26^ To better understand and address potential higher incidence of MIS-C in racial and ethnic minority groups, such as Black, Hispanic or Latino, and Asian or Pacific Islander populations, a more intensive process to ensure collection of timely, complete, and representative data among these populations is needed.^27^

Estimated incidence also varied by age group. Incidence was lowest among persons aged 16 to 20 years. While this aligns with reported characteristics of persons with MIS-C,^28^ this could also be owing to underidentification of MIS-C in this group. Persons reported through national surveillance were most commonly submitted from children’s hospitals, and most sites in Overcoming COVID-19 were children’s hospitals. Persons in this older group might have been more likely to seek care at adult facilities and may not be captured by surveillance.

Similar to our findings, a study by Belay et al^29^ reported geographic differences in cumulative incidence of per-population MIS-C and reported epicurve peaks of MIS-C coinciding with peaks in the COVID-19 pandemic. Our estimation of MIS-C incidence per SARS-CoV-2 infection may help to account for geographic differences in incidence.

### Limitations

This study has limitations. When the race/ethnicity of reported persons with COVID-19 was missing or identified as multiple or other, race/ethnicity was imputed based on the race/ethnicity distribution of the jurisdiction. Imputation was performed for 49% of reported COVID-19 cases. Compared with COVID-19 data with race/ethnicity reported as 1 of the 5 bridged-race categories, the distribution of race/ethnicity of jurisdictions overall tended to be more heavily White and Non-Hispanic, meaning that imputed race/ethnicity for COVID-19 data might have been inaccurately skewed toward this group. Because of the degree of missingness and limited information, imputation based on persons with reported race/ethnicity data exhibited a high degree of variability.

Another limitation is related to the use of previously published month- and age-specific multipliers applied to reported COVID-19 case counts to account for underdetection and to estimate the full burden of SARS-CoV-2 infections.^8^ The uncertainty around these multipliers implies a range of potential estimates of SARS-CoV-2 infections and, in turn, produces a range of estimates in sensitivity analysis for incidence of MIS-C per SARS-CoV-2 infection. These multipliers were summarized at a national level and might overrepresent or underrepresent detection in specific jurisdictions or for particular racial/ethnic groups. Furthermore, because COVID-19 case counts were aggregated by jurisdictions based on month, race/ethnicity, sex, and age group, we were unable to assess MIS-C incidence by other characteristics, such as neighborhood-level poverty or chronic comorbid condition status. In addition, our estimates from only 7 reporting jurisdictions may not be representative of the entire US population. Another limitation is the change in the Overcoming COVID-19 study MIS-C inclusion criteria after June 1; however, by June, testing capability was increased at most study sites, whereas lack of laboratory-confirmed diagnosis during March through May 2020 was likely because of lack of testing capacity.^8^

## Conclusions

This cohort study estimated incidence of MIS-C in select jurisdictions during April to June 2020. These estimates indicated that MIS-C was a rare complication associated with SARS-CoV-2 infection in this cohort overall. The estimates and approach in this study provide baseline data prior to implementation of interventions, such as SARS-CoV-2 vaccination for children, allowing for subsequent monitoring of MIS-C as an outcome, as prevention of SARS-CoV-2 infection would be expected to reduce incidence of MIS-C. Our findings of higher incidence among younger children and among Hispanic or Latino, Black, and Asian or Pacific Islander persons emphasize a need for further study of risk factors for MIS-C.

## Notes

Supplement. **eAppendix 1.** CDC’s National Surveillance Case Report Form **eAppendix 2.** Overcoming COVID-19 Surveillance Registry Case Report Form, Section 1 and 2: Case Definition and Demographic Characteristics **eTable 1.** Multipliers used to estimate number of SARS-CoV-2 infections based on reported persons with COVID-19 **eFigure 1.** Distribution of Reported Persons With COVID-19 in Select Jurisdictions by Month During March to May 2020 **eFigure 2.** Distribution of Reported Persons With MIS-C in Jurisdictions by Select Characteristics During April to June 2020 **eTable 2.** Range of Stratum-Specific Estimates of Incidence of MIS-C per 1 000 000 SARS-CoV-2 Infections in Select Jurisdictions by Jurisdiction, Race/Ethnicity, Sex, and Age Group During April to June 2020
