# Supplementary material for: Incidence of Multisystem Inflammatory Syndrome in Children Among US Persons Infected With SARS-CoV-2
Source: JAMA Netw Open. 2021 Jun 10;4(6):e2116420. doi: 10.1001/jamanetworkopen.2021.16420 (PMC8193431; doi:10.1001/jamanetworkopen.2021.16420)
Supplement: Supplement. — eAppendix 1. CDC’s National Surveillance Case Report Form eAppendix 2. Overcoming COVID-19 Surveillance Registry Case Report Form, Section 1 and 2: Case Definition and Demographic Characteristics eTable 1. Multipliers used to estimate number of SARS-CoV-2 infections based on reported persons with COVID-19 eFigure 1. Distribution of Reported Persons With COVID-19 in Select Jurisdictions by Month During March to May 2020 eFigure 2. Distribution of Reported Persons With MIS-C in Jurisdictions by Select Characteristics During April to June 2020 eTable 2. Range of Stratum-Specific Estimates of Incidence of MIS-C per 1 000 000 SARS-CoV-2 Infections in Select Jurisdictions by Jurisdiction, Race/Ethnicity, Sex, and Age Group During April to June 2020 [file jamanetwopen-e2116420-s001.pdf]

Supplemental Online Content

Payne AB, Gilani Z, Godfred-Cato S, et al; MIS-C Incidence Authorship Group. Incidence of multisystem inflammatory syndrome in children among US persons infected with SARS-CoV-2. *JAMA Netw Open*. 2021;4(6):e2116420. doi:10.1001/jamanetworkopen.2021.16420

eAppendix 1. CDC's National Surveillance Case Report Form

eAppendix 2. Overcoming COVID-19 Surveillance Registry Case Report Form, Section 1 and 2: Case Definition and Demographic Characteristics

eTable 1. Multipliers Used to Estimate Number of SARS-CoV-2 Infections Based on Reported Persons With COVID-19

eFigure 1. Distribution of Reported Persons With COVID-19 in Select Jurisdictions by Month During March to May 2020

eFigure 2. Distribution of Reported Persons With MIS-C in Jurisdictions by Select Characteristics During April to June 2020

eTable 2. Range of Stratum-Specific Estimates of Incidence of MIS-C per 1 000 000 SARS-CoV-2 Infections in Select Jurisdictions by Jurisdiction, Race/Ethnicity, Sex, and Age Group During April to June 2020

This supplemental material has been provided by the authors to give readers additional information about their work.

© 2021 Payne AB et al. *JAMA Network Open.*

eAppendix 1. CDC's National Surveillance Case Report Form[20]

U.S. DEPARTMENT OF HEALTH AND HUMAN SERVICES
CENTERS FOR DISEASE CONTROL AND PREVENTION
ATLANTA, GA 30329

Multisystem Inflammatory Syndrome Associated with COVID-19 Case Report Form

MIS ID *(REQUIRED)*: ________ **Health Department ID:** ________ **NCOV ID** *(if available)*: ________

NNDSS ID *(local_record_id/case id)*: ________ **Tools for CRF data submission to supplement NNDSS case notification/data:** ○ DCIPHER ○ RedCap

Abstractor name: ________ **Date of abstraction:** ___/___/_____

SECTION 1 – INCLUSION CRITERIA

1.1 ☐ Age <21, AND

1.2 ☐ Fever >38.0°C for ≥24 hours, or report of subjective fever lasting ≥24 hours, AND

1.3 ☐ Laboratory markers of inflammation (including, but not limited to one or more; an elevated C-reactive protein (CRP), erythrocyte sedimentation rate (ESR), fibrinogen, procalcitonin, d-dimer, ferritin, lactic acid dehydrogenase (LDH), or interleukin 6 (IL-6), elevated neutrophils, reduced lymphocytes and low albumin, AND

1.4 ☐ Evidence of clinically severe illness requiring hospitalization, with multisystem (≥2) organ involvement *(check all applicable below)*: AND

- **1.4.1** ☐ Cardiac (e.g. shock, elevated troponin, BNP, abnormal echocardiogram, arrhythmia)
- **1.4.2** ☐ Renal (e.g. acute kidney injury or renal failure)
- **1.4.3** ☐ Respiratory (e.g. pneumonia, ARDS, pulmonary embolism)
- **1.4.4** ☐ Hematologic (e.g. elevated D-dimers, thrombophilia, or thrombocytopenia)
- **1.4.5** ☐ Gastrointestinal (e.g. elevated bilirubin, elevated liver enzymes, or diarrhea)
- **1.4.6** ☐ Dermatologic, (e.g. rash, mucocutaneous lesions)
- **1.4.7** ☐ Neurological, (e.g. CVA, aseptic meningitis, encephalopathy)

1.5 ☐ No alternative plausible diagnosis; AND

1.6 ☐ Positive for current or recent SARS-COV-2 infection by (check all applicable below): OR

- **1.6.1** ☐ RT-PCR
- **1.6.2** ☐ Serology
- **1.6.3** ☐ Antigen test

1.7 ☐ COVID-19 exposure within the 4 weeks prior to the onset of symptoms

- **1.7.1** If yes, date of first exposure within the 4 weeks prior : *(MM/DD/YYYY)*: ___/___/_____ ☐ Unknown

SECTION 2 – PATIENT DEMOGRAPHICS

2.1 **State of Residence:** ________

2.2 **Patient zip code**/postal code *(primary residence)*: ________

2.3 **Date of birth** *(MM/DD/YYYY)*: ___/___/_____

2.4 **Sex:** ○ Male ○ Female

2.5 **Ethnicity:** ○ Hispanic or Latino ○ Not Hispanic or Latino ○ Refused or Unknown

2.6 **Race** *(mark all that apply, selecting more than one option as necessary)*:

- **2.6.1** ☐ White
- **2.6.2** ☐ Black or African American
- **2.6.3** ☐ American Indian
- **2.6.4** ☐ Alaska Native or Aboriginal Canadian
- **2.6.5** ☐ Native Hawaiian
- **2.6.6** ☐ Other Pacific Islander
- **2.6.7** ☐ Asian
- **2.6.8** ☐ Other
- **2.6.9** ☐ Refused or Don't know

2.7 **Height:** ________ inches

2.8 **Weight:** ________ lbs

2.9 **BMI:** ________

Comorbidities:

**2.10.1**	Immunosuppressive disorder/malignancy	○ Yes	○ No
**2.10.2**	Obesity	○ Yes	○ No
**2. 10.3**	Type 1 diabetes	○ Yes	○ No
**2. 10.4**	Type 2 diabetes	○ Yes	○ No
**2. 10.5**	Seizures	○ Yes	○ No
**2. 10.6**	Congenital heart disease	○ Yes	○ No
**2. 10.7**	Sickle cell disease	○ Yes	○ No
**2. 10.8**	Chronic lung disease	○ Yes	○ No
**2. 10.9**	Other congenital malformations	○ Yes	○ No
**2. 10.10**	Other *(specify)*: ________		

2.11 Hospital admission date *(MM/DD/YYYY)*: ___/___/_____

- **2.11.1** Number of days in the hospital: ________

2.12 If admitted to the ICU, admission date *(MM/DD/YYYY)*: ___/___/_____

- **2.12.1** Number of days in the ICU: ________

2.13 **Patient outcome:** ○ Died ○ Discharged ○ Still admitted

- **2.13.2** Hospital discharge or death date *(MM/DD/YYYY)*: ___/___/_____

© 2021 Payne AB et al. *JAMA Network Open.*

SECTION 3 – CLINICAL SIGNS AND SYMPTOMS

3.1 Did the patient have preceding COVID-like illness? ○ Yes ○ No

3.1.1 Date of symptom onset *(MM/DD/YYYY)*: ___/___/_____

3.2 Date of symptom onset of MIS *(MM/DD/YYYY)*: ___/___/_____

3.3. Fever ≥ 38.0°C: ○ Yes ○ No

3.3.1 Date of fever onset *(MM/DD/YYYY)*: ___/___/_____

3.3.2 Highest Temperature: _____ °C

3.3.3 Number of days febrile: _____

Signs and symptoms *during present illness*

3.4.1 Cardiac

3.4.1.1	Shock	○ Yes	○ No
3.4.1.2	Elevated troponin	○ Yes	○ No
3.4.1.3	Elevated BNP or NT-proBNP	○ Yes	○ No

3.4.2 Renal

3.4.2.1	Acute kidney injury	○ Yes	○ No
3.4.2.2	Renal failure	○ Yes	○ No

3.4.3 Respiratory

3.4.3.1	Cough	○ Yes	○ No
3.4.3.2	Shortness of breath	○ Yes	○ No
3.4.3.3	Chest pain/tightness	○ Yes	○ No
3.4.3.4	Pneumonia	○ Yes	○ No
3.4.3.5	ARDS	○ Yes	○ No
3.4.3.6	Pulmonary embolism	○ Yes	○ No

3.4.4 Hematologic

3.4.4.1	Elevated D-dimers	○ Yes	○ No
3.4.4.2	Thrombophilia	○ Yes	○ No
3.4.4.3	Thrombocytopenia	○ Yes	○ No

3.4.5 Gastrointestinal

3.4.5.1	Abdominal pain	○ Yes	○ No
3.4.5.2	Vomiting	○ Yes	○ No
3.4.5.3	Diarrhea	○ Yes	○ No
3.4.5.4	Elevated bilirubin	○ Yes	○ No
3.4.5.5	Elevated liver enzymes	○ Yes	○ No

3.4.6 Dermatologic

3.4.6.1	Rash	○ Yes	○ No
3.4.6.2	Mucocutaneous lesions	○ Yes	○ No

3.4.7 Neurological

3.4.7.1	Headache	○ Yes	○ No
3.4.7.2	Altered mental state	○ Yes	○ No
3.4.7.3	Syncope/near syncope	○ Yes	○ No
3.4.7.5	Meningitis	○ Yes	○ No
3.4.7.6	Encephalopathy	○ Yes	○ No

3.4.8 Other

3.4.8.1	Neck pain	○ Yes	○ No
3.4.8.2	Myalgia	○ Yes	○ No
3.4.8.3	Conjunctival injection	○ Yes	○ No
3.4.8.4	Periorbital edema	○ Yes	○ No
3.4.8.5	Cervical lymphadenopathy >1.5 cm diameter	○ Yes	○ No

SECTION 4 – COMPLICATIONS

4.1	**Arrhythmia**	○ Yes	○ No
	If yes:		
4.1.1	Ventricular arrhythmia:	○ Yes	○ No
4.1.2	Supraventricular arrhythmia:	○ Yes	○ No
4.1.3	Other arrhythmia *(specify)*: ____________	○ Yes	○ No
4.2	Congestive heart failure	○ Yes	○ No
4.3	Myocarditis	○ Yes	○ No
4.4	Pericarditis	○ Yes	○ No
4.5	Liver failure	○ Yes	○ No
4.6	Deep vein thrombosis or PE	○ Yes	○ No
4.7	ARDS	○ Yes	○ No
4.8	Pneumonia	○ Yes	○ No
4.9	CVA or stroke	○ Yes	○ No
4.10	Encephalitis or aseptic meningitis	○ Yes	○ No
4.11	Shock	○ Yes	○ No
4.12	Hypotension	○ Yes	○ No

SECTION 5 – TREATMENTS

5.1	Low flow nasal cannula	○ Yes	○ No
5.2	High flow nasal cannula	○ Yes	○ No
5.3	Non-invasive ventilation	○ Yes	○ No
5.4	Intubation	○ Yes	○ No
5.5	Mechanical ventilation	○ Yes	○ No
5.6	ECMO	○ Yes	○ No
5.7	Vasoactive medications (e.g. epinephrine, milrinone, norepinephrine, or vasopressin) *(specify)*: ____________	○ Yes	○ No
5.8	Steroids	○ Yes	○ No
5.9	Immune modulators (e.g. anakinra, tocilizumab) *(specify)*: ____________	○ Yes	○ No
5.10	Antiplatelets (e.g. aspirin, clopidogrel) *(specify)*: ____________	○ Yes	○ No
5.11	Anticoagulation (e.g. heparin, enoxaparin, warfarin) *(specify)*: ____________	○ Yes	○ No
5.12	Dialysis	○ Yes	○ No
5.13	First IVIG	○ Yes	○ No
5.14	Second IVIG	○ Yes	○ No

© 2021 Payne AB et al. *JAMA Network Open.*

SECTION 6 – STUDIES

6.1 Blood Test Results

**6.1.1**	Fibrinogen	Highest value: ______	units: ______		O Low	O Normal	O High
**6.1.2**	CRP	Highest value: ______	units: ______		O Low	O Normal	O High
**6.1.3**	Ferritin	Highest value: ______	units: ______		O Low	O Normal	O High
**6.1.4**	Troponin	Highest value: ______	units: ______		O Low	O Normal	O High
**6.1.5**	BNP	Highest value: ______	units: ______		O Low	O Normal	O High
**6.1.6**	NT-proBNP	Highest value: ______	units: ______		O Low	O Normal	O High
**6.1.7**	D-dimer	Highest value: ______	units: ______		O Low	O Normal	O High
**6.1.8**	IL-6	Highest value: ______	units: ______		O Low	O Normal	O High
**6.1.9**	Serum White blood count	Highest value: ______	Lowest value : ______	units: ______			
**6.1.10**	Platelets	Highest value : ______	Lowest value : ______	units: ______			
**6.1.11**	Neutrophils	Highest value: ______	Lowest value : ______	units: ______			
**6.1.12**	Lymphocytes	Highest value: ______	Lowest value : ______	units: ______			
**6.1.13**	Bands	Highest value: ______	Lowest value : ______	units: ______			

6.2 CSF Studies

**6.2.1**	White blood count	Highest value : ______	Lowest value : ______	units: ______
**6.2.2**	Protein	Highest value : ______	Lowest value : ______	units: ______
**6.2.3**	Glucose	Highest value : ______	Lowest value : ______	units: ______

6.3 Urinalysis

**6.3.1**	Urine White blood count	Highest value : ______	Lowest value : ______	units: ______

6.4 Echocardiogram (check if seen on ANY echocardiogram)

- **6.4.1** ☐ Not done
- **6.4.2** ☐ Normal results
- **6.4.3** ☐ Coronary artery aneurysms
  - **6.4.3.1** Max coronary artery Z-score: ______
- **6.4.4** ☐ Coronary artery dilatation
- **6.4.5** ☐ Cardiac dysfunction (decreased function), specify type:
  - **6.4.5.1** ☐ left ventricular dysfunction
  - **6.4.5.2** ☐ right ventricular dysfunction
- **6.4.6** ☐ Pericardial effusion
- **6.4.7** ☐ Pleural effusion
- **6.4.8** ☐ Mitral regurgitation, specify type: O mild O moderate O severe
- **6.4.9** ☐ Other *(specify)*: ______

6.5 Date of first test showing coronary artery aneurysm or dilatation *(MM/DD/YYYY)*: ___/___/_____

6.6 Abdominal imaging ☐ Ultrasound ☐ CT O Not done

- **6.6.1** ☐ Normal
- **6.6.2** ☐ Mesenteric lymphadenopathy
- **6.6.3** ☐ Free fluid
- **6.6.4** ☐ Other *(specify)*: ______

6.7 Chest imaging ☐ Chest x-ray ☐ CT O Not done

- **6.7.1** ☐ Normal
- **6.7.2** ☐ Pneumonia
- **6.7.3** ☐ Atelectasis
- **6.7.4** ☐ Pleural effusion
- **6.7.5** ☐ Other *(specify)*: ______

SARS-COV-2 testing

6.8 RT-PCR: O Positive O Negative O Not done
6.8.1 If performed, date *(MM/DD/YYYY)*: ___/___/_____

6.9 Antigen: O Positive O Negative O Not done
6.9.1 If performed, date *(MM/DD/YYYY)*: ___/___/_____

6.10 IgG: O Positive O Negative O Not done
6.10.1 If performed, date *(MM/DD/YYYY)*: ___/___/_____

6.11 IgM: O Positive O Negative O Not done
6.11.1 If performed, date *(MM/DD/YYYY)*: ___/___/_____

6.12 IgA: O Positive O Negative O Not done
6.12.1 If performed, date *(MM/DD/YYYY)*: ___/___/_____

© 2021 Payne AB et al. *JAMA Network Open.*

eAppendix 2. Overcoming COVID-19 Surveillance Registry Case Report Form, Section 1 and 2: Case Definition and Demographic Characteristics

SECTION 1 CASE DEFINITION
(for each surveillance time period)

Case Definition Through May 31st, 2020

Inclusion Criteria

- Hospitalization at a participating site in a pediatric unit
- ≤ 25 years old
- Disease is suspected of being related to SARS-CoV-2:
    - SARS-CoV-2 positive PCR AND/OR
    - SARS-CoV-2 positive antibody test AND/OR
    - Meets MIS-C criteria (see below) and hospitalized March 15th - May 31st, 2020

Exclusion Criteria

Not hospitalized OR >25 years old OR no suspected association with SARS-CoV-2

Multisystem Inflammatory Syndrome in Children (MIS-C) Criteria

Inclusion Criteria (must have all 3)

- Fever ≥38 ºC (100.4 ºF) for ≥ 24 hours, or report of subjective fever lasting ≥ 24 hours, **AND**
- Laboratory markers of inflammation (including but not limited to one or more of the following: elevated C-reactive protein (CRP), erythrocyte sedimentation rate (ESR), fibrinogen, procalcitonin, d-dimer, ferritin, lactic acid dehydrogenase (LDH), or interleukin 6 (IL-6), elevated neutrophils, reduced lymphocytes and low albumin), **AND**
- Clinical evidence of severe hospitalized illness including multi-organ (≥ 2) involvement based on clinical judgement from record review, discharge diagnosis, laboratory or diagnostic tests:
    - Cardiac (e.g. shock, elevated troponin, BNP, abnormal echocardiogram, arrhythmia)
    - Respiratory (e.g. pneumonia, ARDS, pulmonary embolism)
    - Renal (e.g. acute kidney injury or renal failure)
    - Gastrointestinal (e.g. abdominal pain, vomiting, diarrhea, elevated bilirubin, or elevated liver enzymes)
    - Neurologic, (e.g. CVA, aseptic meningitis, encephalopathy)
    - Hematologic (e.g. elevated D-dimers, thrombophilia, or thrombocytopenia)
    - Dermatologic (e.g. rash, erythema, peeling)
    - Fulfill full or partial criteria for complete or incomplete Kawasaki disease
    - Other (specify):________________________

Exclusion criteria: Other likely microbial or other cause, including bacterial sepsis, staphylococcal or streptococcal shock syndromes.

Case Definition June 1, 2020 through August 12, 2020

Inclusion Criteria

- Hospitalization at a participating site in a pediatric unit
- ≤ 21 years old
- SARS-CoV-2 positive PCR or SARS-CoV-2 positive antibody test
- Symptoms of COVID-19 or MIS-C that prompted test (not done as a screening test for an elective procedure or visit)

Exclusion Criteria

© 2021 Payne AB et al. *JAMA Network Open.*

- Negative (PCR and antibody) or no testing for SARS-CoV-2 (COVID-19)

Case Definition August 13, 2020 forward

Inclusion Criteria

- Hospitalization at a participating site in a pediatric unit
- < 21 years old (exclude if 21 years on admit)
- SARS-CoV-2 PCR, antigen, or antibody positive
- Meets one or more of the following:
  - Admitted to the ICU or stepdown unit for COVID-related complications AND/OR
  - Meets CDC criteria for MIS-C (see above)

Exclusion Criteria

- Negative (PCR, antigen, and antibody) or no testing for SARS-CoV-2 (COVID-19)

© 2021 Payne AB et al. *JAMA Network Open.*

SECTION 2 PATIENT DEMOGRAPHICS

2.1 Date of birth (MM/DD/YYYY): ____/____/______

2.2 Is patient <1 year of age on admission?
☐ Yes ☐ No

2.2.1 If yes, born prematurely? (Born before 37 weeks of gestation)
☐ No
☐ Unknown
☐ Yes:
2.2.1.1 Number of weeks of gestation at birth: _____ weeks
2.2.1.2 Corrected gestational age at time of illness: _____ weeks

2.3 Gender:
☐ Male
☐ Female

2.4 Ethnicity:
☐ Hispanic or Latino
☐ Not Hispanic or Latino
☐ Refused or Unknown

2.5 Race (*mark all that apply, selecting more than one option as necessary*):

2.5.1	White or White-Hispanic	☐ Yes	☐ No
2.5.2	Black or African American or Black-Hispanic	☐ Yes	☐ No
2.5.3	American Indian	☐ Yes	☐ No
2.5.4	Alaska Native or Aboriginal Canadian	☐ Yes	☐ No
2.5.5	Native Hawaiian	☐ Yes	☐ No
2.5.6	Other Pacific Islander	☐ Yes	☐ No
2.5.7	Asian (Asian Indian, Chinese, Filipino, Japanese, Korean, Vietnamese)	☐ Yes	☐ No
2.5.8	Other	☐ Yes	☐ No
	2.5.8.1 Please list: ________________________________		
2.5.9	Refused or Don't know	☐ Yes	☐ No

2.6 First 4 digits of patient zip code/postal code (primary residence): ___ ___ ___ ___

2.7 Insurance:
☐ Private
☐ Self-pay
☐ U.S. Government (e.g. Medicaid)
☐ Unknown

© 2021 Payne AB et al. *JAMA Network Open.*

eTable 1. Multipliers Used to Estimate Number of SARS-CoV-2 Infections Based on Reported Persons With COVID-19

Adapted from Reese et al. 2020[8]

Month	Age Group	Multiplier (95% Confidence Interval)
**March**	≤5 Years	191.0 (95.2–412.3)
	6–10 Years	222.6 (109.9–484.0)
	11–15 Years	222.6 (109.9–484.0)
	16–20 Years	32.2 (18.1–65.6)
**April & May**	≤5 Years	15.7 (9.8–26.6)
	6–10 Years	16.4 (10.1–27.9)
	11–15 Years	16.4 (10.1–27.9)
	16–20 Years	7.1 (5.0–10.1)

© 2021 Payne AB et al. *JAMA Network Open.*

eFigure 1. Distribution of Reported Persons With COVID-19 in Select Jurisdictions by Month During March to May 2020

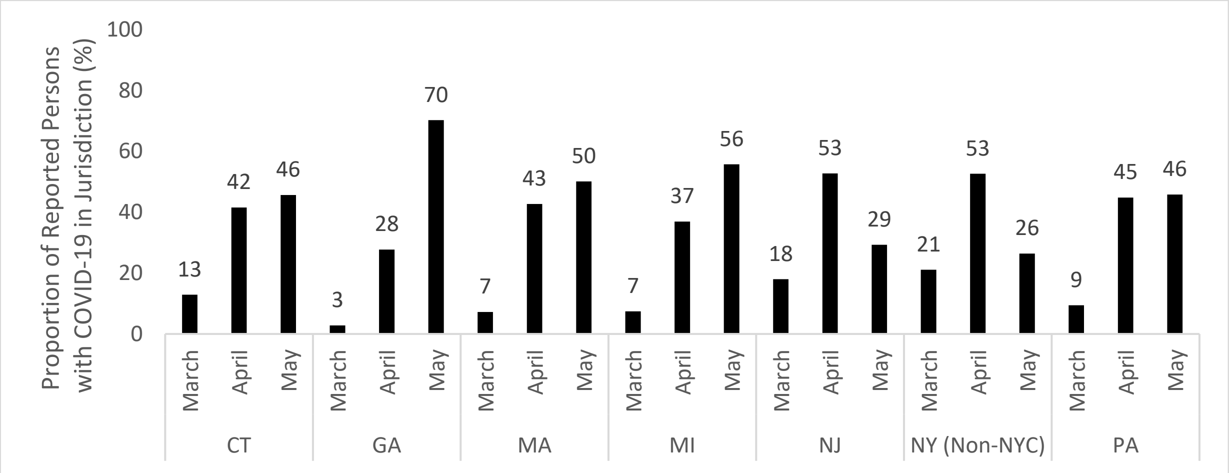

Abbreviations: CT, Connecticut; GA, Georgia; MA, Massachusetts; MI, Michigan; NJ, New Jersey; NY (Non-NYC), New York (Non-New York City); PA, Pennsylvania

© 2021 Payne AB et al. *JAMA Network Open.*

eFigure 2. Distribution of Reported Persons With MIS-C in Jurisdictions by Select Characteristics During April to June 2020

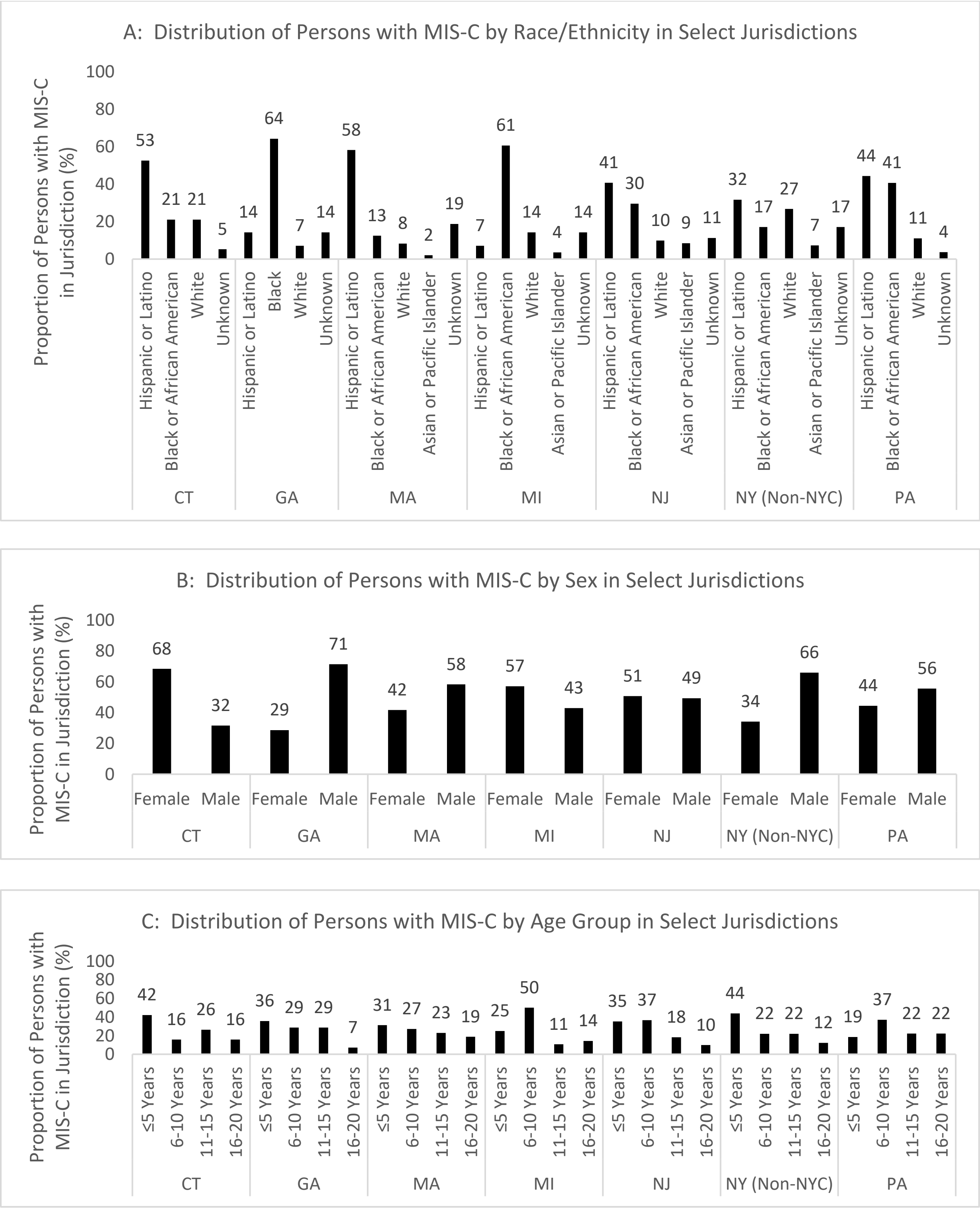

© 2021 Payne AB et al. *JAMA Network Open.*

Abbreviations: CT, Connecticut; GA, Georgia; MA, Massachusetts; MI, Michigan; NJ, New Jersey; NY (Non-NYC), New York (Non-New York City); PA, Pennsylvania

© 2021 Payne AB et al. *JAMA Network Open.*

eTable 2. Range of Stratum-Specific Estimates of Incidence of MIS-C Per 1 000 000 SARS-CoV-2 Infections in Select Jurisdictions by Jurisdiction, Race/Ethnicity, Sex, and Age Group During April to June 2020

	Adjusted[A] MIS-C Incidence per million persons with reported COVID-19 [B] (95% CI[C])	Adjusted[A] MIS-C Incidence per million SARS-CoV-2[B,D] Infections (95% CI[B]CI[C]) (Low[E])	Adjusted[A] MIS-C Incidence per million SARS-CoV-2[B,D] Infections (95% CI[C]) (Mid[F])	Adjusted[A,G,H] MIS-C Incidence per million SARS-CoV-2[B,D] Infections (95% CI[C]) (High[I])
**Jurisdiction**				
Connecticut	8,807 (5,397–14,371)	191 (117–312)	358 (219–585)	625 (383–1,020)
Georgia	4,014 (2,223–7,246)	142 (78–256)	246 (136–444)	402 (222–727)
Massachusetts	6,812 (4,805–9,658)	194 (137–276)	352 (248–498)	593 (419–840)
Michigan	12,762 (8,319–19,578)	345 (223–534)	627 (405–969)	1,063 (689–1,641)
New Jersey[J]	11,087 (8,342–14,735)	169 (126–226)	331 (248–443)	599 (448–800)
New York (Non-New York City)	5,371 (3,743–7,708)	70 (48–100)	138 (96–198)	252 (175–362)
Pennsylvania	7,661 (5,052–11,616)	193 (127–293)	355 (234–540)	608 (400–923)
**Race/Ethnicity[G]**				
White	2,910 (2,040–4,152)	58 (41–83)	110 (77–156)	192 (135–274)
Black or African American	15,380 (12,093–19,561)	329 (257–422)	616 (481–790)	1,073 (839–1,372)
Hispanic or Latino	9,947 (7,935–12,470)	257 (204–324)	467 (371–588)	794 (631–999)
Asian or Pacific Islander	7,309 (3,982–13,414)	170 (91–319)	315 (169–589)	544 (292–1,012)
**Sex[H]**				
Female	7,252 (5,630–9,342)	167 (129–215)	309 (240–399)	533 (414–688)
Male	7,866 (6,160–10,044)	173 (135–222)	323 (252–413)	559 (437–716)
**Age Group**				
<6 years	14,292 (10,782–18,944)	229 (172–305)	444 (333–591)	798 (600–1,061)
6–10 Years	19,624 (14,892–25,859)	318 (240–420)	613 (464–811)	1,105 (836–1,460)
11–15 Years	7,293 (5,236–10,159)	116 (83–161)	224 (160–312)	404 (289–563)
16–20 Years	1,591 (1,071–2,363)	99 (67–148)	164 (110–243)	250 (169–372)

© 2021 Payne AB et al. *JAMA Network Open.*

[A]Adjusted using Poisson regression, with jurisdiction, race/ethnicity, sex, and age group in the model
[B]Reflects estimate after imputation of race/ethnicity for N=16,129 persons with COVID-19 reported from jurisdictions
[C]CI: confidence interval
[D]SARS-CoV-2 infections estimated by applying age- and month-specific multipliers[8] to reported COVID-19 case counts
[E]'Low' incidence estimate corresponds to use of upper bound of confidence interval around multiplier estimate
[F]'Mid' incidence estimate corresponds to use of point estimate for multiplier
[G]N = 32 persons with MIS-C with other/unknown race/ethnicity excluded from analyses involving race/ethnicity, including adjusted estimates
[H]N = 188 reported persons with COVID-19 with other/unknown sex excluded from analyses involving sex, including adjusted estimates
[I]'High' incidence estimate corresponds to use of lower bound of confidence interval around multiplier estimate
[J]NJ suppressed stratum-specific reported COVID-19 case counts of <5; these were assumed to be 1 for analysis

Abbreviations: MIS-C: multisystem Inflammatory Syndrome in Children; SARS-CoV-2: severe acute respiratory syndrome coronavirus 2

© 2021 Payne AB et al. *JAMA Network Open.*
